# Low Back Pain among Medical Students in Belgrade (Serbia): A Cross-Sectional Study

**DOI:** 10.1155/2018/8317906

**Published:** 2018-02-06

**Authors:** Isidora Vujcic, Nemanja Stojilovic, Eleonora Dubljanin, Nebojsa Ladjevic, Ivana Ladjevic, Sandra Sipetic-Grujicic

**Affiliations:** ^1^Institute of Epidemiology, Faculty of Medicine, University of Belgrade, Belgrade, Serbia; ^2^Institute of Microbiology and Immunology, Faculty of Medicine, University of Belgrade, Belgrade, Serbia; ^3^Institute of Urology and Nephrology, Clinical Center of Serbia, Belgrade, Serbia; ^4^Clinic for Obstetrics and Gynecology, Clinical Center of Serbia, Belgrade, Serbia

## Abstract

**Aim:**

To examine the prevalence of low back pain, to identify self-perceived triggers of low back pain, and to investigate the impact of perceived pain on the daily activities and mood among medical students.

**Methods:**

This cross-sectional study enrolled 459 fourth year students at the Faculty of Medicine in Belgrade during December 2014. The anonymous questionnaire was used for data collection. In data analysis, the chi-square test and *t*-test were used.

**Results:**

The lifetime prevalence of low back pain was 75.8%, 12-month prevalence 59.5%, and point prevalence 17.2%. Chronic low back pain was experienced by 12.4% of the students. Both the lifetime (*p*=0.001) and the 12-month (*p*=0.001) low back pain prevalence rates were significantly higher among female medical students. Mental stress during an exam period (*p*=0.001), sitting at the university (*p*=0.002), fatigue (*p*=0.043), improper body posture (*p*=0.005), and lack of exercise (*p*=0.001) as self-perceived triggers of low back pain were significantly more often reported by female students, compared to males. Regarding daily functioning, the experience of low back pain mostly affects students sleeping (14.6%) and walking (12.0%).

**Conclusions:**

The prevalence of LBP is high among Belgrade medical students and significantly affects their everyday functioning.

## 1. Introduction

Low back pain (LBP) is one of the most common health problems and affects people of all ages, from children to the elderly [1, 2]. According to the Global Burden of Disease 2010 Study, the prevalence and burden of LBP are very high throughout the world [3]. Out of the 291 conditions studied, LBP was found to have the sixth highest burden and to cause more disability globally than any other condition [3]. In 2010, the age-standardized point prevalence was highest in Western Europe (15%) and North Africa/Middle East (14.8%). The age-standardized point prevalence in Central Europe, including Serbia, was 11.5% (12.6% in males and 10.3% in females) and ranked the fifth place [3]. However, it is estimated that over 80% of the population will experience an episode of LBP at some time during life, and that about 18% of the population experience LBP at any given moment [4].

The main risk factors for LBP are age, gender, obesity, psychosocial factors (stress, anxiety, and depression), level of education, occupational factors, decreased flexibility and mobility of muscles, hypermobility, competition sports, type and way of carrying and transporting weight, postural habits, level of physical activity, smoking, and domestic factors such as watching TV and computer/videogame [5, 6]. LBP often begins in childhood, and the prevalence rate for adolescents approaches that seen in adults [7]. Epidemiological studies have shown an increase in LBP in children, teenagers, and young adults [8], but studies exhibit great variability in prevalence rates [7]. It is challenging to compare the prevalence of LBP between populations and over time due to methodological heterogeneity across studies such as the age of the sample, the sample size, the definition of LBP, the LBP recall period, the strategy for extracting data, the methodology used, and difficulties in obtaining true population estimates [6, 7].

Some studies reported a high prevalence of LBP among medical students [9–11]. Due to highly demanding curriculum during the studies, medical students are exposed to stress, sedentary lifestyle, and long hours on hospital wards and clinics which may lead to the high prevalence of LBP in this population. Also, the presence of LBP can affect medical students' productivity, their attendance at lectures and medical training, and therefore their future career. Higher prevalence of LBP was observed among students with five or more semesters, suggesting that advanced students who most often are exposed to practical activities are at increased risk for disease [12].

The aims of this study were to examine the prevalence of LBP (lifetime, 12-month, and point prevalence) among Belgrade clinical medical students, to identify self-perceived triggers of LBP, and to investigate the impact of perceived pain on their daily activities and mood.

## 2. Participants and Methods

### 2.1. Study Participants

A cross-sectional study was conducted in December 2014 among fourth year students at the Faculty of Medicine in Belgrade. The study was approved by Institutional Review Board of the Faculty of Medicine, University of Belgrade. Students were recruited before the start of compulsory practical sessions at the Institute of Epidemiology. The purpose of the study and questionnaire's content were described to the students. A total 459 of all 533 fourth year medical students completed the questionnaire fully (response rate was 86.12%).

### 2.2. Questionnaire

Each student had to fill out an anonymous questionnaire that included questions regarding the following: demographic characteristics (age, gender, and place of living), the prevalence of LBP (lifetime, 12-month, and point prevalence) and chronic LBP, self-perceived triggers of LBP (stress at the university during the exam period, sitting at the university, uncomfortable mattress, and improper body posture), the impact of pain on daily functioning (sleeping, walking, and everyday activities), and the impact of pain on mood (anger and anxiety). In order to assess self-perceived triggers of LBP and the impact of pain on daily functioning and mood, a numerical rating scale was used ranging from zero (no impact) to six (extremely strong impact). To facilitate data analysis, we collapsed responses to no impact, small/moderate impact, and strong/extremely strong impact. For students, LBP was defined as pain in the area between the inferior margin of the 12th rib and inferior gluteal folds [3, 13]. The lifetime prevalence was estimated as the proportion of respondents who had ever suffered LBP at some point in their lives. The 12-month prevalence referred to the presence of LBP in the last year, and the point prevalence referred to the presence of LBP at the moment of filling out the questionnaire (currently). We classified LBP as chronic if it was present for more than 12 weeks.

### 2.3. Statistical Analysis

Statistical differences between male and female students were tested using the chi-square test and *t*-test. A *p* value of <0.05 was considered statistically significant.

## 3. Results

### 3.1. Prevalence of Low Back Pain

The mean age of study participants was 22.46 years (SD ± 0.95), and two-thirds (66.0%) were female students. Out of 459 investigated students, 75.8% reported LBP at some point in their lives, 59.5% in the last 12 months, and 17.2% of them were suffering from LBP at the moment they were surveyed (Table 1). Chronic LBP reported 12.4% of the students. The lifetime and 12-month prevalence of LBP were significantly higher among female than male medical students, while the point prevalence was similar in both groups.

### 3.2. Self-Perceived Subjective Triggers of LBP

**Figure 1:**
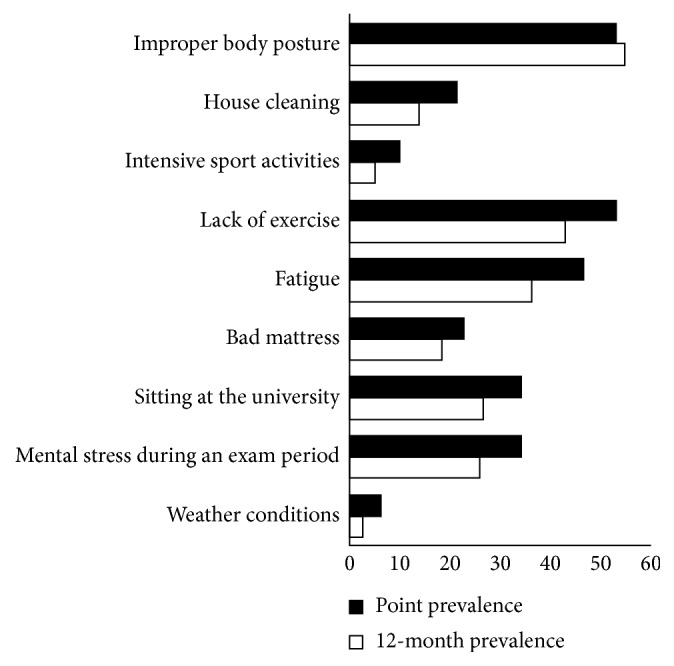
Self-perceived subjective triggers with strong/extremely strong impact on low back pain among medical students.

The most often cited self-perceived subjective triggers with strong/extremely strong impact on the point and 12-month LBP prevalence were the following, respectively: improper body posture (53.3% versus 54.9%), lack of exercise (53.2% versus 42.9%), and fatigue (46.8% versus 36.3%). Mental stress during an exam period and sitting at the university as potential triggers for currently present LBP reported about one-third of the investigated students (34.2%) (Figure 1). Other reported triggers were bad mattress, house cleaning, intensive sport activities, and weather conditions.

Statistically significant gender difference regarding self-perceived subjective triggers of LBP was found for mental stress during an exam period, sitting at the university, fatigue, lack of exercise, and improper body posture which were reported more often by female students (Table 2).

### 3.3. The Impact of LBP on Everyday Functioning and Mood

**Figure 2:**
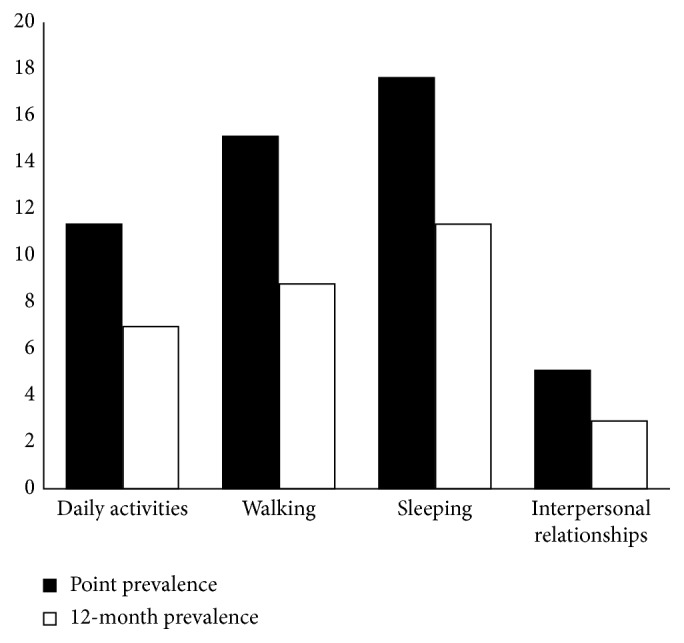
The impact of low back pain on medical students' everyday functioning.

Regarding everyday functioning, LBP mostly affects students sleeping (14.6%) and walking (12.0%) (Figure 2), while personal interrelationships are less likely affected by the presence of LBP. Similar findings were observed for the presence of LBP currently and in the previous 12 months.

**Figure 3:**
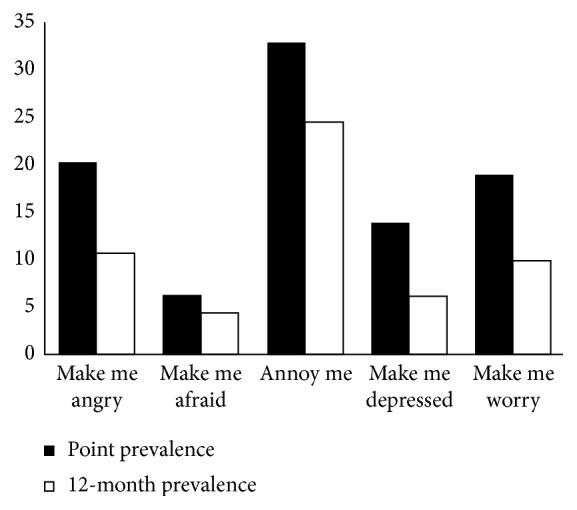
The impact of low back pain on medical students' mood.

The most medical students cited that the presence of LBP currently and in the last year annoys them and makes them angry and worry (Figure 3).

## 4. Discussion

According to the results of our study, the point prevalence of LBP among Belgrade medical students was 17.2%, 12-month prevalence 59.5%, and lifetime prevalence 75.8%. These findings are consistent with other studies, indicating that the point prevalence is less than the period prevalence and both are less than the lifetime prevalence [1, 7, 14]. The 12-month prevalence of LBP was 47.5% among students in India [9], 46.1% among Malaysian students [10], and 53.4% among Austrian medical students [15]. In a Brazilian study, which included medical and physiotherapy students, the lifetime prevalence of LBP only among medical students was 73.4%, 12-month prevalence 59.9%, and point prevalence 9.2%, while physiotherapy students reported a higher prevalence of LBP when compared with the medical students in all measures [12]. The lifetime and 12-month prevalence of LBP observed in our study were similar to those of the Brazilian study, but the point prevalence of LBP in our study was significantly higher than that in the Brazilian study. Higher point prevalence in our study may be explained by the fact that, in our study, fourth year medical students who were on clinical training participated and higher prevalence of LBP was observed among older medical students, who are exposed to practical activities as mentioned earlier. In the Brazilian study, more than half of the medical students (51.2%) were preclinical, until the fourth semester. The Chinese study, conducted among fourth year medical students, as in our study, revealed a 12-month LBP prevalence of 40.1% and a point prevalence of 17.9% [11].

Female medical students in our study reported significantly higher 12-month and lifetime prevalence, compared to male students, while the point prevalence of LBP was similar in both genders; in fact, the rate was lower in female students. According to studies conducted among children and adolescents, girls have higher prevalence rates of LBP than boys [16, 17]; therefore, the lifetime LBP prevalence is expected to be higher in female students. Regarding studies conducted among medical students, most of the studies did not find the significant difference in the 12-month LBP prevalence between genders including the Chinese study [11], the study from India [9], and the Malaysian study as well, which investigated the prevalence of musculoskeletal pain including LBP too [10]. The Brazilian study compared the prevalence of LBP among medical and physiotherapy students, and the Austrian study compared the prevalence of LBP among the medical students and random sample of physical education students, and therefore they were not focused on the gender difference of LBP among medical students. In contrast, the Australian study found that female medical students, compared to males, 1.8 times more often reported LBP [18].

The most often cited self-perceived subjective triggers with strong/extremely strong impact on LBP occurrence in our study were improper body posture, lack of exercise, and fatigue. The study conducted among medical students in India [9] also showed that abnormal body posture was one of the factors independently associated with LBP. Although lack of exercise was reported as a risk factor for LBP in some studies [19, 20], there is still conflicting evidence on the potential association between physical activity and low back pain in both general population and school children [21]. Fatigue is a common complaint among medical students, and the study of Tanaka et al. [22] found that stress and coping styles were associated with severe fatigue in medical students. Medical students in our study reported fatigue as the third most often trigger of LBP.

However, female medical students, in comparison with male students, significantly more often reported mental stress during an exam period, sitting at the university, fatigue, lack of exercise, and improper body posture as potential triggers for LBP. In addition, the 12-month prevalence of LBP was significantly higher among female students. Taking into account that LBP is a multidimensional disorder, with physical, lifestyle, and psychosocial factors related to its development and maintenance [23], a possible explanation for these findings includes that female students, in comparison with males, are more emotionally sensitive and feel fatigued more easily [24]. Also, females have greater sensitivity to painful stimuli and lower pain thresholds compared to males [25]. The study conducted among Australian adolescents indicates that being female and sitting posture are related factors for LBP, and that greater proportion of female participants than male participants were complaining of back pain that was made worse by sitting [23], which is very similar to the results of our study. The sex relationship with posture may be related to sex differences in the shape of the pelvis, greater back muscle endurance in women, sex-specific motor activation patterns, inappropriate furniture at the university and hospitals, behavioral issues, and other social factors such as anxiety and depression [23].

Numerous studies showed that multiple domains of psychosocial functioning are affected by LBP experience such as social relationships, self-esteem, mood and affect, social roles, family duties, life satisfaction, and independence in satisfying one's own needs [26]. In our study among medical students, LBP mostly affects their sleeping quality and walking. The study conducted at four universities in Poland indicates that LBP interferes with or limits daily activities of the students, such as sitting, standing, or physical activity [27], and according to this study, LBP influences sleeping in 17.7% of participants and walking in 12.8% which is very similar to the results of our study. Also, LBP experience affects medical students' mood, and most of them make worry. Therefore, it is necessary to organize physical education classes at least three times a week or daily at the faculty, as well as educational programs that theaches practical information about body mechanics and how to protect the back.

Our study has several limitations. The study participants were fourth year medical students on clinical training, and therefore, it was not possible to make a comparison among students of all six study years. Also, the survey has been undertaken in only one institution and should be expanded to include medical students from other universities in the country. Data about the experience of LBP, potential triggers, and impact of pain on daily functioning and mood were based on self-reports, and information bias is probably present. We did not collect data about BMI, smoking habits, and physical activity which future research should take into account.

## 5. Conclusions

The prevalence of LBP is high among Belgrade medical students and significantly affects their everyday functioning and mood. Female students have significantly higher 12-month and lifetime prevalence of LBP, compared to males.

## Figures and Tables

**Table 1:** Prevalence of low back pain among male and female medical students.

Characteristics	All students	Males	Females	*p* value
	(*n*=459)	(*n*=156)	(*n*=303)	
	Number (%)	Number (%)	Number (%)	
Age (*x* ± SD) (years)	22.46 ± 0.95	22.45 ± 1.02	22.46 ± 0.91	0.859^∗^
Place of living				
Belgrade	179 (39)	61 (39.1)	118 (38.9)	1.000^∗∗^
Others	280 (61)	95 (60.9)	185 (61.1)	
Lifetime prevalence of LBP				
Yes	348 (75.8)	103 (66.0)	245 (80.9)	0.001^∗∗^
The 12-month prevalence of LBP				
Yes	273 (59.5)	74 (47.4)	199 (65.7)	<0.001^∗∗^
Point prevalence of LBP				
Yes	79 (17.2)	28 (17.9)	51 (16.8)	0.795^∗∗^
Chronic LBP (pain persists 12 weeks or longer)				
Yes	57 (12.4)	15 (9.6)	42 (13.9)	0.232^∗∗^

^∗^According to the *t*-test, ^∗∗^according to the chi-square test, LBP: low back pain.

**Table 2:** Self-perceived subjective triggers connected with occurrence of low back pain among medical students regarding their gender.

Factors	Males	Females	*p* value^∗^
	(*n*=156)	(*n*=303)	
	Number (%)	Number (%)	
Weather conditions			
Small/moderate impact	16 (15.5)	61 (24.9)	0.153
Strong/extremely strong impact	5 (4.9)	12 (4.9)	
Mental stress during an exam period			
Small/moderate impact	32 (31.1)	85 (34.7)	<0.001
Strong/extremely strong impact	20 (19.4)	91 (37.1)	
Sitting at the university			
Small/moderate impact	34 (33.0)	77 (31.4)	0.002
Strong/extremely strong impact	23 (22.3)	99 (40.4)	
House cleaning			
Small/moderate impact	39 (37.9)	82 (33.5)	0.555
Strong/extremely strong impact	18 (18.4)	57 (23.2)	
Bad mattress			
Small/moderate impact	37 (35.9)	82 (33.4)	0.862
Strong/extremely strong impact	36 (25.3)	68 (27.8)	
Fatigue			
Small/moderate impact	31 (30.1)	56 (22.9)	0.043
Strong/extremely strong impact	33 (32.0)	114 (46.5)	
Lack of exercise			
Small/moderate impact	32 (20.5)	65 (21.5)	<0.001
Strong/extremely strong impact	43 (27.6)	136 (44.8)	
Improper body posture			
Small/moderate impact	27 (26.2)	51 (20.8)	0.005
Strong/extremely strong impact	51 (49.5)	163 (66.5)	

^∗^According to the chi-square test.
